# Recent increase in the occurrences of Christmas typhoons in the Western North Pacific

**DOI:** 10.1038/s41598-021-86814-x

**Published:** 2021-04-01

**Authors:** Joseph Basconcillo, Il-Ju Moon

**Affiliations:** 1 Typhoon Research Center, Jeju National University, Jeju, South Korea; 2 Department of Science and Technology, Philippine Atmospheric, Geophysical, and Astronomical Services Administration, Quezon City, Philippines

**Keywords:** Atmospheric science, Climate change, Climate sciences

## Abstract

To imply the gravity of their impact on Christmas celebration, the term Christmas typhoon recently became more popular to refer to tropical cyclones (TC) in the Western North Pacific (WNP) during its less active season. The past 9 years from 2012 to 2020 saw more than 70% (210%) increases in Christmas typhoon occurrences in the WNP (Philippines). Furthermore, Mindanao Island, which is located in southern Philippines, has experienced an unprecedented 480% increase in TC passage in the same period. Here we show that the detected recent increase in Christmas typhoons are mainly associated with the shift of the Pacific Decadal Oscillation to its positive phase in early 2010s, which led to favorable changes in the large-scale environment for TC development such as higher relative vorticity, anomalous low-level westerlies, warmer sea surface temperatures in the central Pacific, and extended WNP subtropical high. We also found that the poleward shift of the Intertropical Convergence Zone and possibly, the recent recovery of the Siberian High contributed to such increased occurrences. As opposed to the more active TC season, there is a wide research gap during the less active season. We aim to fill in this knowledge gap to gain better insights on TC risk reduction.

## Introduction

**Figure 1:**
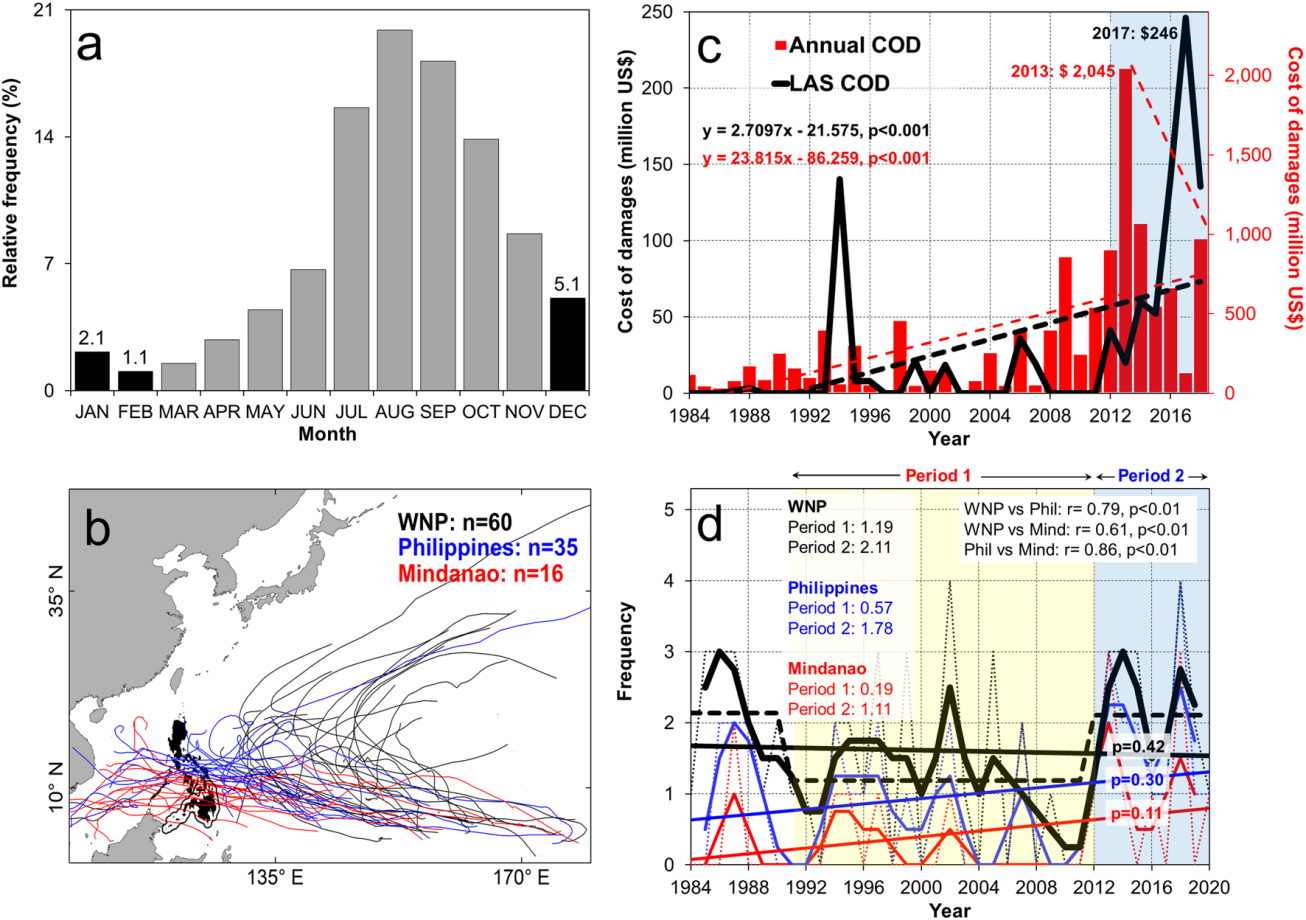
Climatology of Christmas typhoons. (**a**) Monthly tropical cyclone (TC) relative frequency in the Western North Pacific (WNP). The black (gray) bars indicate the less active season (more active season). (**b**) TC tracks during the less active season (LAS) in the WNP (black), in the Philippines (blue), and in Mindanao Island (red). The black shade (line) indicates the location of the Philippines (Mindanao Island). (**c**) Cost of damages (COD) associated with TCs in the Philippines. The red (black) bar (line) indicates the annual (LAS) cost of damages. The significant trend of cost of damages during LAS (black) and in annual terms (red) increased (decreased) in 2012. (**d**) Mean annual Christmas typhoon frequency in the WNP (black), in the Philippines (blue), and in Mindanao (red). The black dashed lines indicate the regime shifts in the WNP during Period 1 (yellow box) and Period 2 (blue box). In (**d**), the inset statistics show the correlation of the timeseries with each other, and the mean TC frequency during Period 1 and Period 2, respectively. The *p* values of the trends are shown on top of their respective trend lines. In (**b**), the map is plotted using ArcGIS 10.1 (https://www.esri.com).

In a year, there are more than 20 tropical cyclones (TC) that form in the Western North Pacific (WNP) where its more active season peaks from June to November and its less active season (LAS) runs from December to February (DJF; Fig. 1a). From 1984 to 2020, there were 60 TCs during the LAS that developed in the WNP. Among them, 35 TCs (58%) crossed within 200 km of the Philippines. The island of Mindanao, which does not usually experience TC passages because of its southern location, observed 16 TCs (27%) in the same period (Fig. 1b).

“Christmas typhoon” is popularly referred to TC occurrences usually from December to February^1–5^ to denote their impacts on top of dampening the world’s longest Christmas celebration^6^. Hereafter, we will use Christmas typhoons to refer to TC occurrences during the said season. Accordingly, one of the earliest reports where Christmas typhoon is used was during the passage of Typhoon Quantico in the Philippines in December 1918^7^.

The estimated total cost of damages associated with Christmas typhoons in the Philippines accounted to US$ 654.2 million where the highest amount of US$ 246 million is recorded in 2017^8^ (Fig. 1c). While the annual cost of damages appear to decrease after 2013, which is after the passage of Typhoon Haiyan, this cannot be said true during the LAS because of its significant increase in the cost of damages (*p* < 0.001; two-tailed) since 2012. The said upsurge in the cost of damages is corroborated by the significant increase in Christmas typhoons in WNP, in the Philippines, and in Mindanao since 2012 (*p* < 0.001, two-tailed; Fig. 1d). It is noteworthy that 11 out of the 16 Christmas typhoons that passed over Mindanao from 1984 to 2020 are recorded in a span of 9 years from 2012 to 2020, which is one of the reasons why there is an increasing cost of damages during the LAS in the Philippines.

There is an apparent lack of literature on TC variability during the LAS^9,10^ as opposed to the wide array of studies during the more active season. We attribute this knowledge gap to the limited number of TCs during the LAS and to their regional clustering in the WNP and in the Philippines. Moreover, the large-scale environment in the WNP during the LAS is marked by the absence of summer monsoon flow replaced by northeasterlies, deeper region of high relative vorticity and WNP subtropical high, cooler sea surface temperatures (SST), reduced cloudiness, and weakened low-level convergence, which are considerably less favorable for TC development^11,12^ (Supplementary Fig. S1a–c). The limited number of observed TCs and unfavorable conditions for TC development during the LAS prompt TC research to focus on the more active season, combine Christmas typhoons with the preceding months, and/or cluster all TCs together, including the LAS, on an annual or semi-annual scale^9,10,13,14^, which essentially dilutes the variability that is inherent to Christmas typhoons and consequentially, renders a false sense of security for hazard risk reduction^15^. In the advent of changing climate and the apparent lack of knowledge thereof, it is not surprising that the occurrences of Christmas typhoons are being associated with the new normal of Christmas celebration in the Philippines^16^.

Because Christmas typhoons are poorly understood phenomena in the field of TC research, it becomes necessary to ask what influences their variability. In the succeeding discussion, we argue that Christmas typhoons are driven by interannual to multi-year variability but its recent increased occurrences are mainly attributed to the shifts of the Pacific Decadal Oscillation in the early 2010s to its positive phase. We also discuss the other sources of variability of Christmas typhoons that are not previously described in known literature.

## Results

### Increase in Christmas typhoon occurrences

Previous reports are in conflict whether there is a long term trend of Christmas typhoons due to their differences in the use of timescale and study period^9,10^. Here we show that there is no significant long term trend in Christmas typhoon frequency in the WNP, in the Philippines, and in Mindanao from 1984 to 2020 (Fig. 1d). However, using a climate regime shift index^17,18^, a significant downward regime shift is detected in 1991 (Period 1) until an upward shift in 2012 (Period 2; *p* < 0.05, two-tailed) in the WNP, in the Philippines, and in Mindanao. In the WNP (Philippines), the mean TC frequency during Period 1 is 1.19 (0.57) while there are 2.11 (1.78) TCs during Period 2, which translates to more than 70% (210%) increase. In Mindanao, the mean TC frequency increased from 0.19 during Period 1 to 1.11 during Period 2, which indicates greater than 480% increase. Given that the Christmas typhoon frequency in the WNP is significantly correlated with that in the Philippines (r = 0.79, *p* < 0.000, two-tailed) and in Mindanao (r = 0.61, *p* < 0.000, two-tailed), we used the timeseries in the WNP in the succeeding discussion.

One concern that has to be resolved is whether the small number of years in Period 2 warrants effectively significant difference from Period 1. The power analysis of the compared independent means shows that for a given alpha of 0.05 the probability of obtaining a significant difference between Period 1 sampled at 20 years and Period 2 sampled at 9 years is 97% of the time while the risk of committing a Type II error while such probability is false is 3% of the time (Supplementary Table S1). Furthermore, a Cohen’s d effect size^19^ that is greater than 0.8 indicates that the magnitude of the difference between the two periods is effectively apart from each other. The negative sign of the effect size implies the direction of the effect, which denotes that the mean of Period 1 is effectively smaller than the mean of Period 2. While we recognize the limited number of TC occurrences during the LAS, the 37-year timeseries is deemed sufficient because the timeseries has no significant long term trend and it also covers the reported reliable time period of analysis for TC research, which starts in 1984^20^.

**Figure 2:**
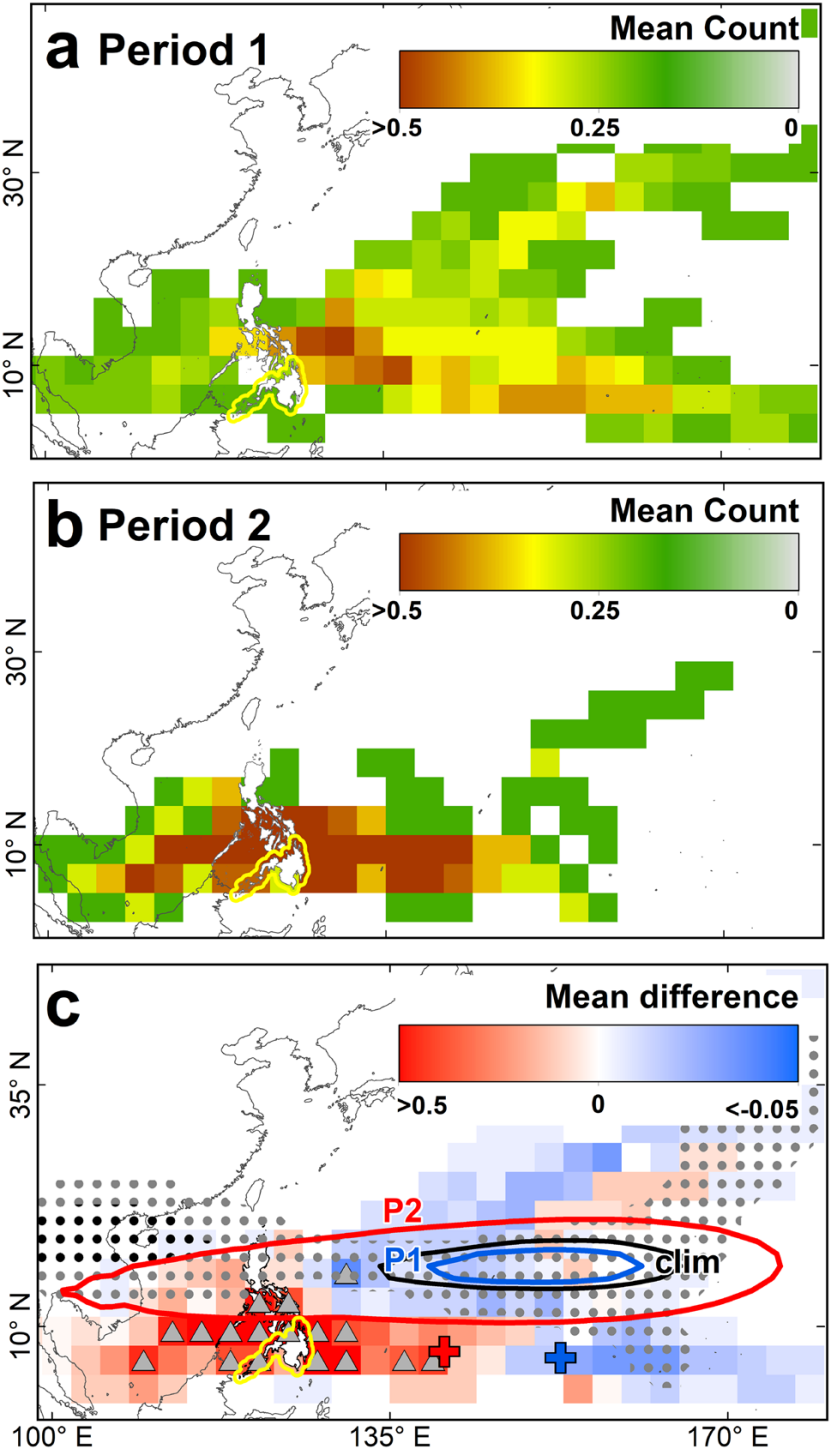
Christmas typhoon passages in the Western North Pacific. Mean TC passage frequency during Period 1 (**a**) and Period 2 (**b**). (**c**) Difference in mean TC passage frequency between Period 2 and Period 1. The gray triangles indicate significant difference in mean TC passage frequency at *p* < 0.05 level. The black (gray) dots represent the significant difference in geopotential height at *p* < 0.01 (*p* < 0.05) level, respectively. The red (blue) cross denotes the mean TC genesis point during Period 2 (Period 1). The red and blue contours represent the location of the WNP subtropical high (= 5873 gpm) during the Period 2 (P2) and Period 1 (P1) while the black contour shows its climatological location (clim). The yellow line indicates the location of Mindanao Island. In (**a**–**c**), the maps are plotted using ArcGIS 10.1 (https://www.esri.com).

The mean Christmas typhoon passage frequency during Period 1 and Period 2 (Fig. 2a,b) show an increased typhoon passages towards the Philippines, particularly toward its southern portions during the Period 2 (Fig. 2c). Such finding is supported by a wider and extended WNP subtropical high flanking around 10° N of the Philippines, which directs Christmas typhoon passages toward the southern Philippines. The WNP subtropical high regulates TC tracks and their variability in the WNP^21^.

**Figure 3:**
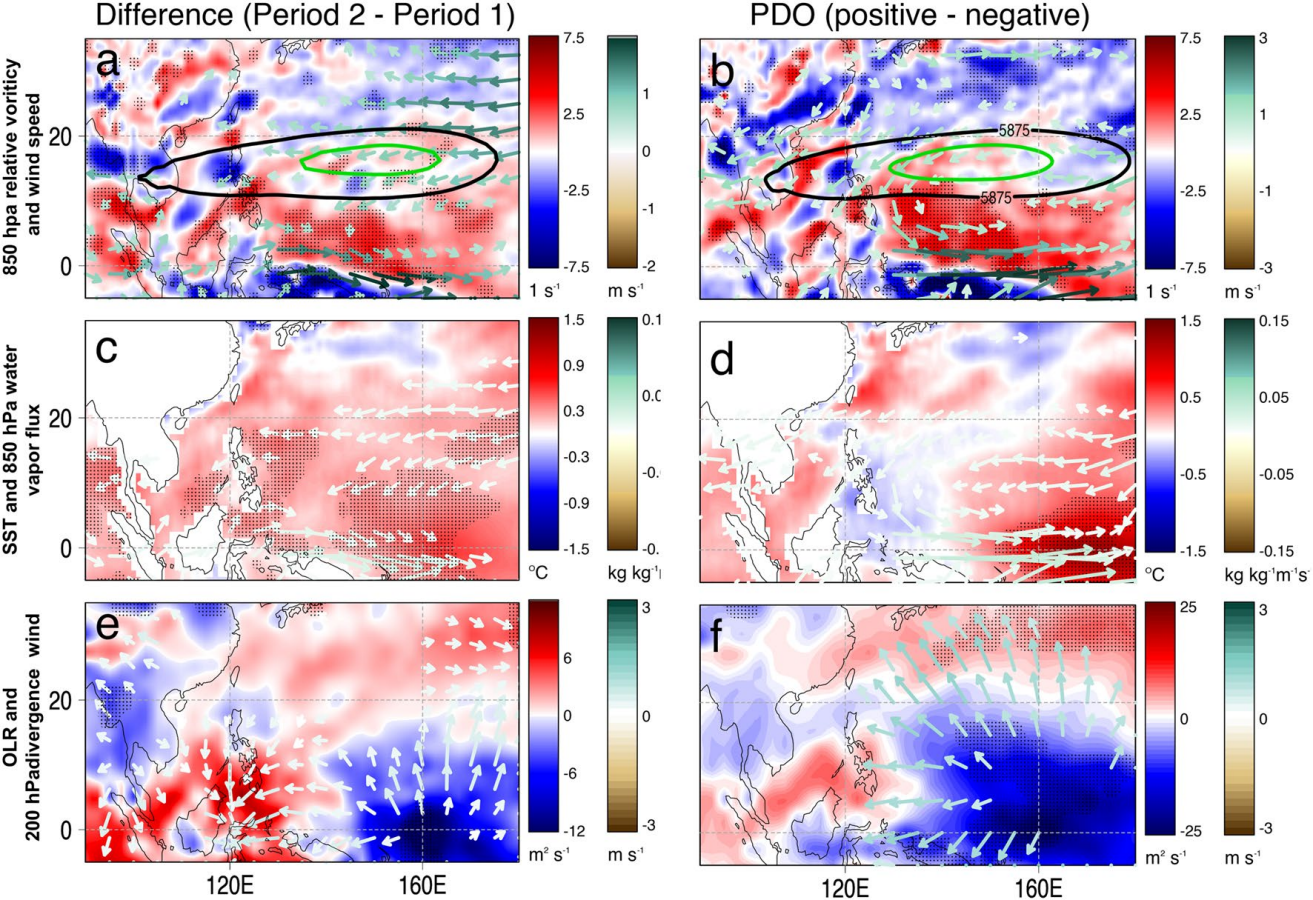
Composite analysis of indicated variables. Left panel, Composite difference between Period 2 and Period 1 showing 850 hPa relative vorticity (shaded) and wind speed (vector) (**a**), sea surface temperature (shaded) and 850 hPa water vapor flux (**c**) and outgoing longwave radiation (shaded) and 200 hPa divergence wind (vector) (**e**). Right panel, Similar to left panel but showing the difference between positive and negative PDO years (**b**–**f**). The magnitude of difference in the shaded and vector variables is shown in their respective scale bars. The black dots indicate significant difference at *p* < 0.05 level, two-tailed. In (**a**–**b**), the 850 hPa relative vorticity is multiplied with 1,000,000 for scaling while the black (green) contour shows the location of the WNP subtropical high during Period 2 (Period 1) and positive (negative) PDO phase, respectively. The maps are plotted using GrADS v2.2.1 (http://opengrads.org/).

The composite difference between Period 2 and Period 1 shows significant changes in the large-scale environmental conditions that are favorable for Christmas typhoon genesis, which include higher 850 hPa relative vorticity, anomalous low-level westerlies, warmer sea surface temperatures (SST), stronger 850 hPa water vapor fluxes, and anomalous convective and low-level convergent activities (Fig. 3a,c,e; *p* < 0.05, two-tailed). An increase in water vapor fluxes in a humid environment is more favorable for TC formation compared to a dry environment^22,23^. The negative outgoing longwave radiation indicates the presence of anomalous convective activities and low-level convergence to the east of the Philippines that are directed by the extended WNP subtropical high towards the Philippines.

In absence of the summer monsoon trough, the convergence of the anomalous equatorial westerlies with easterlies provides background higher low-level cyclonic vorticity, which results to the formation of anomalous cyclonic circulation in the warm SST region of the central Pacific (around 160° E). In this sense, the higher relative vorticity bands the convective anomalies together to form pre-existing disturbances that are necessary for TC genesis^11,12^.

Moreover, the warm SSTs translate to higher air-sea temperature difference, which results to higher low-level water vapor fluxes from the sea to the atmosphere that reduce the entrainment of dry air and further enhances the convective anomalies^22,23^. The enhancement of precipitation from the higher water vapor fluxes results to the strengthening of cyclonic perturbation in a vortex whose strength is relative weak, which gives way to TC development^24^.

### Influence of SST-based modes of variability

The El Niño Southern Oscillation (ENSO) is a dominant interannual mode of SST variability in the equatorial Pacific that tends to peak during the boreal winter^25^. A number of previous studies have established the linkage between ENSO and permutations of TC metrics such as frequency, intensity, duration, etc.^26,27^. Even though these studies mostly focused on the more active season and other timescale variations, it remains surprising that there is insufficient literature on the linkage between ENSO and Christmas typhoons.

It is previously reported that the positive phase of ENSO is unfavorable for Christmas typhoon formation because the warm SST anomalies located to the east of the Philippines are relatively small and the background atmospheric conditions are also unfavorable for increased upward motion^9^, which could suppress pre-existing convective activities. However, it is widely thought that the interannual variations of ENSO, particularly its warm phase, are favorable for active tropical convection and associated stronger upward motion that are precursors for TC development^25–28^.

There are several known ENSO flavors^29^ and their influence to TC development varies with spatial and temporal scale of analysis. Here we show that among the ENSO flavors, the Central Pacific ENSO index (CP ENSO)^30^ has the only significant correlation with Christmas typhoon frequency (r = 0.34, *p* = 0.049, two-tailed; Supplementary Figs. S2a, S3a). Albeit the marginal correlation, a warm CP ENSO condition prompts higher relative vorticity, stronger low-level westerlies, higher water vapor fluxes, enhanced cloudiness in the central Pacific, and an extended WNP subtropical high, which directs TCs towards the Philippines (Supplementary Fig. S4a–c).

In longer timescale, the Pacific Decadal Oscillation (PDO) is one of the prevailing multiyear modes of SST variability characterized by warm SST anomalies north of 20° N in the Pacific during its warm phase and a converse effect during its cool phase. While the PDO exists in all months, its maximum seasonal amplitude is typically observed during the boreal winter^31,32^. We used the DJF PDO in our analysis. We found a strong significant relationship between PDO^21^ and Christmas typhoon frequency (r = 0.73, *p* < 0.000, two-tailed; Supplementary Fig. S3b). Meanwhile, we did not find significant correlation between Christmas typhoons and the Interdecadal Pacific Oscillation (r = − 0.17), which is another known mode of multiyear SST variability in the Pacific Ocean. Perhaps, this is because the Interdecadal Pacific Oscillation has an oscillation period of 40–60 years^33^, which is beyond our covered analysis period.

To highlight patterns associated with PDO, we plotted the composite difference of large-scale environment during positive and negative PDO years (Fig. 3b,d,f). There are considerable similarities between the changes in environmental conditions in Period 2 and during positive PDO years, which include higher relative vorticity, prominent anomalous cyclonic circulation to the east of the Philippines, anomalous westerlies, wetter environment, warmer SST anomalies, anomalous convective activities, stronger upward motion, and an extended WNP subtropical high. Such changes, as previously described, are favorable conditions for TC genesis.

### Influence of the other modes during the boreal winter

The ITCZ is typically associated with high low-level vorticity and high low-level relative humidity; therefore, it becomes a favorable area for TC genesis^34^. During the boreal winter, the ITCZ in the Indo-Pacific Warm Pool region (0°–22° N, 80°–170° E) that is characterized by prominent zonally elongated precipitation band is located around 5–10° N (Supplementary Fig. S5a).

Using the Empirical Orthogonal Function (EOF) transform, we found that the principal component of the second leading EOF mode of precipitation in the Indo-Pacific Warm Pool region is highly correlated with Christmas typhoon frequency (r = 0.67, *p* < 0.01, two-tailed; (Supplementary Fig. S5d) and its highest amount of precipitation has strong resemblance (Supplementary Fig. S5b) to the location of convective anomalies in the central Pacific during the Period 2 (Fig. 3e) and in a positive PDO phase (Fig. 3f), which suggests the influence of the warm pool ITCZ to Christmas typhoons. We quantified the warm pool ITCZ using its northern latitude as a metric of its locational shifts and its intensity (Supplementary Fig. S6a,b). Of these two ITCZ metrics, the warm pool ITCZ latitude has significant correlation with WNP Christmas typhoon frequency (r = 0.54, *p* = 0.001, two-tailed) while the ITCZ intensity has no significant correlation. In addition, the mean ITCZ location has also shifted northwards during the Period 2 (Supplementary Figs. S5a, S6a), which is perhaps because a more poleward ITCZ is indicative of wider and poleward favorable background environment for TC genesis^35^.

Another unexpected research gap is the limited literature on the relationship between the Siberian High and Christmas typhoons given that the former is another dominant mode during the boreal winter. Most of the existing studies only relate the Siberian High with the Asian winter monsoon^36^ and associated precipitation in the WNP and in the Philippines^37^, and it remains unknown how the Siberian High influences the variability of Christmas typhoons.

We did not find significant correlation between the Siberian High intensity and Christmas typhoons (r = 0.16). However, the correlation map of Siberian High intensity^38,39^ with the 400 hPa geopotential height from 1984 to 2020 illustrate its atmospheric teleconnection patterns (Supplementary Fig. S7a). To highlight the teleconnection pattern that are related with the Siberian High, we used 400 hPa level because the positive vertical vorticity associated with the Tibetan High reaches its maximum at 500 hPa and disappears at 400 hPa^40^. The Siberian High is teleconnected with a high pressure region that flanks over mainland Southeast Asia and appears to be conjoined with the WNP subtropical high. This begs the question whether the Siberian High is responsible for the more significant difference in geopotential height in the mainland Southeast Asia (shown in black dots in Fig. 2c) and the more extended WNP subtropical high during the Period 2 (Fig. 2c).

When the Siberian High is more intense, the East Asian subtropical jetstream becomes significantly stronger^38^ (Supplementary Fig. 7a–c). The warm southerlies from the mainland Southeast Asia are stalled by the strengthened East Asian subtropical jetstream to flank towards Taiwan and northern Philippines, which means that the East Asian subtropical jetstream serves as a conduit between the high pressure region in mainland Southeast Asia and the WNP subtropical high.

Meanwhile, a positive PDO phase corresponds to a weak Aleutian low^41^ and the downstream effect of a positive ENSO phase also translates to a deeper and weaker Aleutian Low^42^. A weak Aleutian Low means that the ridge of a high pressure system from the North Pacific is wider and extended southwards^43^. When the extended ridge of high pressure system in the North Pacific conjoins with the stalled high pressure in mainland Southeast Asia associated with the Siberian High, both separate systems act as a barrier directing TCs towards southern Philippines (Fig. 2c).

We calculated the partial correlation of the Siberian High with Christmas typhoon frequency while controlling for CP ENSO given that the ENSO is more dominant in the tropics and that it might dilute the relationship of the Siberian High with Christmas typhoons. We found higher magnitude of partial correlations of the high-pressure region over the mainland Southeast Asia and more prominent East Asian subtropical jetstream. Although the partial correlation of Christmas typhoon frequency with Siberian High remains insignificant (Table 1), the demonstrated partial correlation patterns may indicate the influence of the Siberian High to directing the passages of Christmas typhoons towards southern Philippines.

**Table 1:** Partial correlation coefficients of Christmas typhoons and selected climate indices when controlling for PDO and CP ENSO.

	Christmas typhoon frequency
**PDO**	
CP ENSO	0.10 (0.604)
Siberian High intensity	− 0.23 (0.23)
ITCZ EOF2	0.23 (0.226)
ITCZ Latitude	0.11 (0.569)
**CP ENSO**	
PDO	**0.69 (0.000)**
Siberian High intensity	− 0.082 (0.668)
ITCZ EOF2	**0.5 (0.005)**
ITCZ Latitude	**0.42 (0.022)**

Values in bold and inside parentheses indicate their corresponding significant partial correlation and *p* value using two-tailed distribution test.

Meanwhile, a declining trend in the intensity of Siberian High is reported from 1978 to 2011^38^ while a contrasting report on increasing trend is found since 1990^39^. We show that the partial correlation of the Siberian High over the mainland Southeast Asia from 1984 to 2020 (1984 to 2011) becomes significant (insignificant) (Supplementary Fig. S7b,c), which possibly indicates that the recent recovery in the Siberian High intensity may have possibly exerted influence to the recent increase in Christmas typhoon passages towards the Philippines. In contrast with the boreal summer when the intensity of the WNP summer monsoon heavily influences TC variations in the WNP, we found that the East Asian winter monsoon intensity^26^ does not have significant correlation with the variations of Christmas typhoons in the WNP (r = 0.1).

Considering that the PDO has the highest correlation with Christmas typhoon frequency, we calculated the partial correlation of Christmas typhoon frequency with CP ENSO, Siberian High intensity, the principal component of the second leading EOF mode of warm pool ITCZ precipitation, and the warm pool ITCZ latitude while controlling for PDO (Table 1). The partial correlations of CP ENSO, SHI, and warm pool ITCZ latitude and the principal component of the second leading EOF mode of warm pool ITCZ precipitation became lower and insignificant.

Likewise, we performed partial correlation analysis of the same variables while controlling for CP ENSO. The resulting partial correlations of Christmas typhoon frequency with PDO, principal component of the second leading EOF mode of warm pool ITCZ precipitation, and the warm pool ITCZ latitude became lower but they remained significant, which implies that the PDO is the more dominant forcing on Christmas typhoons than the CP ENSO and that the recent shift of the PDO to its positive phase, more active convection and warmer SSTs in the central Pacific, and the northward shifts of the ITCZ resulted to the recent increase in the occurrences of Christmas typhoons.

On the other hand, intraseasonal modes such as the Madden–Julian Oscillation (MJO) could also influence Christmas typhoons considering that it becomes more active, more symmetric along the equator, and achieves its seasonal maximum during the boreal winter^44^. The MJO is an eastward-propagating intraseasonal system of tropical convective activities that travels through its different phases every 30–60 days. The MJO is nearest to the Philippines and WNP during its Phases 5–6. In 2018, when four Christmas typhoons developed in the WNP, the MJO during its Phases 5–6 was near its record strength^45^, which implies that the above normal MJO activity at that time may have concurrently influenced above normal Christmas typhoon development.

## Discussion

Since 2012, a growing number of reports have identified with climate change or new normal of living^5^ as a framework in explaining the recent occurrences of Christmas typhoons, especially in the Philippines. Without the sufficient understanding of Christmas typhoons, such leaping statements, which include blaming climate change as the culprit of the increased Christmas typhoons occurrences, are inevitable. This also goes without saying that the people were disconcerted because of these knowledge gaps as they celebrate the holiday season.

**Figure 4:**
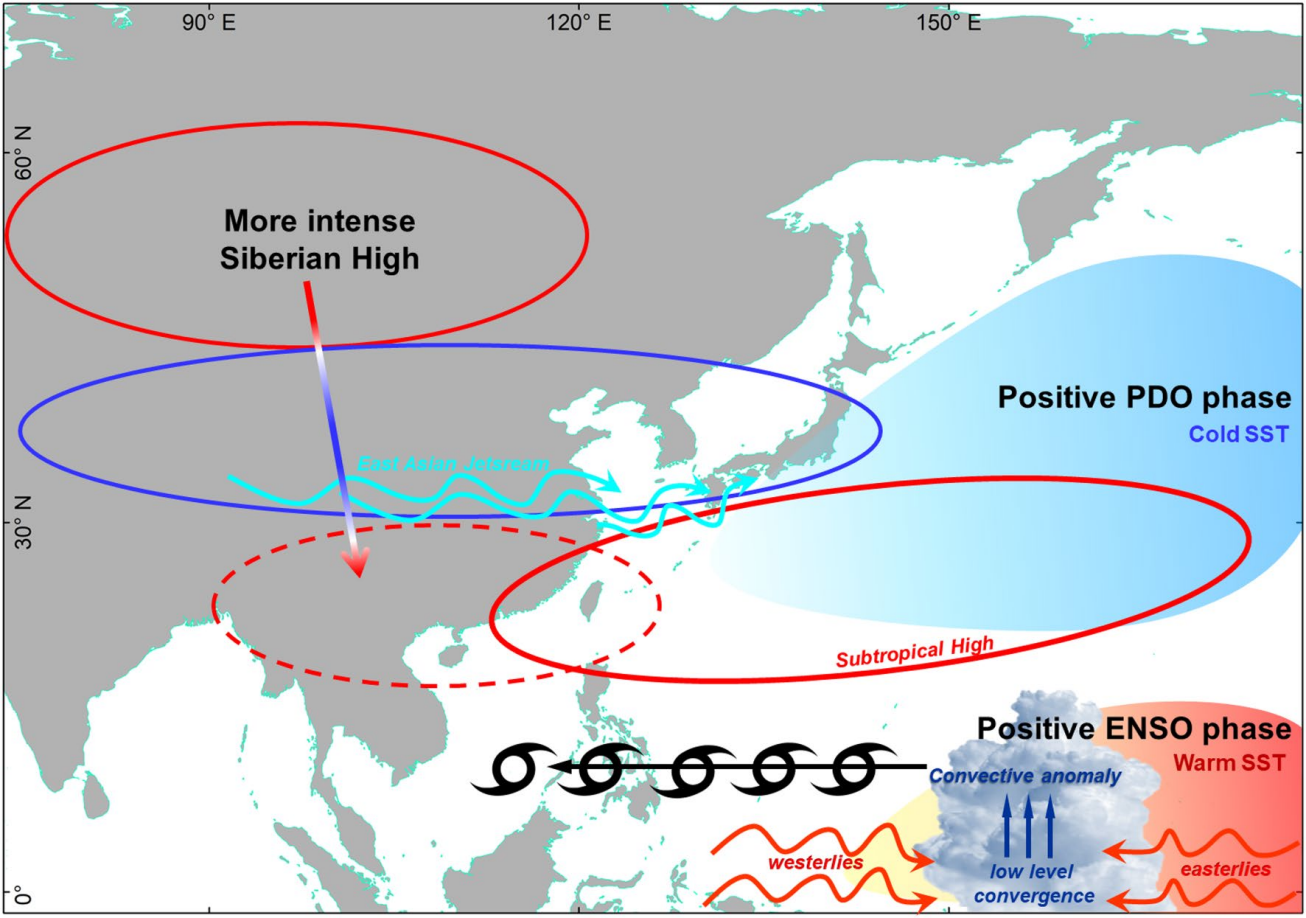
Influences to Christmas typhoons. A positive PDO phase is coincident with a positive ENSO phase, which is characterized by cold (warm) SST anomalies in the north (tropics). The anomalous low-level westerlies provide background cyclonic vorticity and converge with low-level easterlies leading to the formation of convective anomalies. The higher air-sea temperature difference results to more water vapor fluxes from the sea and reduced entrainment of dry air that enhances the convective anomalies. The westward extension of the WNP subtropical high directs the tropical cyclones toward the Philippines. Meanwhile, a more intense Siberian High corresponds to stronger subtropical jetstream and highlighted by its stronger teleconnection with a stalled high pressure system flanking over the mainland Southeast Asia and extending towards the northern Philippines. The schematic is drawn using Microsoft PowerPoint (https://www.microsoft.com/).

Driven by these knowledge deficiencies, our study has identified that the recent increase in Christmas typhoon occurrences is mostly related to the shift of the PDO to its positive phase in the early 2010s (Fig. 4). A positive PDO phase is associated with a favorable background for TC genesis such as warm SST anomalies that results to higher water vapor fluxes, convergence of anomalous low-level westerlies and easterlies providing background cyclonic vorticity to the formation of convective anomalies, which is eventually directed by an extended WNP subtropical high towards the Philippines and Southeast Asia. We have also demonstrated that the warmer SSTs and active tropical convection in the central Pacific and the northward shift in the latitudinal position of the ITCZ during the Period 2 have contributed to the recent increase in Christmas typhoons. Meanwhile, the recent recovery of the Siberian High may have indirectly influenced the recent increase in Christmas typhoons.

In addition to the absence of significant increasing trend in Christmas typhoons, we did not find significant correlation between Christmas typhoon frequency and global mean temperature during the LAS. We also found that the Christmas typhoons have a low frequency periodicity of 3–4 years and high frequency variability of 13–16 years (Supplementary Fig. S8), which implies that the Christmas typhoons remain to be influenced by seasonal to multiyear modes of variability and that it has predictable horizons, which could provide new insights on the predictability of Christmas typhoons with sufficient lead time in support of hazard risk reduction.

## Methods

### Reanalysis and tropical cyclone dataset

We used the Japanese Reanalysis 55-year project in our analysis^46^. The TC best track data are obtained from the International Best Track Archive for Climate Stewardship version 4 from 1984 to 2020^47^. Our period of analysis covers the period 1984–2020 parallel to the reported reliable period of TC in the WNP^20^. Moreover, only named TCs with maximum sustained winds that are greater than or equal to 35 knots (~ 17 ms^−1^) were considered in our analysis. If indicated, the TC frequency timeseries is filtered using the 1-2-1 technique^48^.

We classified the seasonal TC activity in the Western North Pacific into its more active season from March to November and its less active season (LAS) from December of previous year to February of the following year. For example, the total typhoon frequency in 2018 is the total number of typhoons in December 2017 and January to February 2018. We mark December as the start of LAS because the large-scale atmospheric circulation in the WNP has largely shifted to the dominant northeasterly winds as compared with November.

The associated cost of damages with tropical cyclones in the Philippines is obtained from the National Disaster Risk Reduction and Management Council Operations Center of the Department of National Defense-Philippines. We converted the cost of damages from Philippine Peso to US Dollar where US $1 = Philippine Peso 50.

### Climate indices

The climate indices used in our analysis are defined as follows:

- Niño 3 index—areal-averaged DJF SST anomalies at 5° S–5° N, 210°–270° E^29^
- Niño 4 index—areal-averaged DJF SST anomalies at 5° S–5° N, 160°–210° E^29^
- Central Pacific ENSO: Normalized Niño 4 index minus Normalized Niño 3 index^30^
- Pacific Decadal Oscillation index—leading principal component of DJF SST north of 20° N in the Pacific Ocean^29^
- Interdecadal Pacific Oscillation index—difference in SST anomalies over the central and equatorial Pacific, and the northwest and southwest Pacific^29^
- East Asian Winter Monsoon index—difference in normalized areal-averaged DJF mean sea level pressure at 45°–60° N, 70°–120° E minus 30°–50° N, 140°–190° E and 20° S–10° N, 110°–160° E^36^
- Siberian High intensity—areal-averaged DJF mean sea level pressure at 45°–65° N, 80°–120° E^38,39^
- Niño West index—areal-averaged DJF SST anomalies at 0°–10° N, 130°–150° E^49^
- ITCZ latitude—the northern latitude of zonal-averaged precipitation (80°–180° E) at 9 mm day^−1^.
- ITCZ intensity—the area-averaged precipitation in the Indo-Pacific warm pool region (0°–22° N, 80°–170° E)

In the composite analysis, a positive (negative) PDO and CP ENSO year is defined as a year that is greater than or equal (less than or equal) to 1 (− 1) standard deviation of their climatology from 1984 to 2020. The positive PDO years include 1987, 2002, 2013, 2015, 2016, and 2017 while the negative PDO years are 1992, 1993, 1998, 2008, 2009, 2010, 2011, and 2012. The positive CP ENSO years are 1988, 1991, 1995, 2002, 2003, 2005, 2007, 2010, and 2015 while the negative CP ENSO years include 1984, 1989, 1998, 1999, 2000, 2001, 2008, 2009, 2011, and 2012.

### Statistical tests

The Pearson’s Correlation is the selected method of measuring correlation and partial correlation in our study where their significance is tested using two-tailed distribution.

The composite difference used t-test statistic with two-tailed distribution for significance testing. The power analysis of the sampled means and calculation of effect size using Cohen’s d^19^ were performed to test the power of significance of difference of means between the two periods. The significance of trend in timeseries was tested using the Mann–Kendall Test. The periodicity of Christmas typhoons was based on Paul wavelet transform method.

Using the Rodionov algorithm^13,14^, we calculated the regime shift of the 1-2-1 filtered Christmas typhoon frequency with a cut-off length of 5, Hubert’s Parameter of 2, and significance level at *p* < 0.05 level.

## Supplementary Information

Supplementary Informations.

## Data Availability

The reanalysis data products used in the analysis are available for download from their respective websites.
