# Supplementary material for: Recent increase in the occurrences of Christmas typhoons in the Western North Pacific
Source: Sci Rep. 2021 Apr 1;11:7416. doi: 10.1038/s41598-021-86814-x (PMC8016983; doi:10.1038/s41598-021-86814-x)
Supplement: Supplementary file 1 — Supplementary Informations. [file 41598_2021_86814_MOESM1_ESM.docx]

**Recent Increase in the Occurrences of Christmas Typhoons in the Western North Pacific**

**Joseph Basconcillo^1, 2, +^, Il-Ju Moon^1,*, +^**

^1^Typhoon Research Center, Jeju National University, Jeju, South Korea

^2^Philippine Atmospheric, Geophysical, and Astronomical Services Administration, Department of Science and Technology, Quezon City, Philippines

^*^ ijmoon@jejunu.ac.kr

^+^these authors contributed equally

**Supplementary Information**

This Supplementary Information includes:

Supplementary Table S1

Supplementary Figs. S1-S8

**Supplementary Table S1. Parameters of the power analysis of difference in sampled means and effect size of tropical cyclone frequency during Period 1 and Period 2.**

| **Parameter** | **Period 1** | **Period 2** |
| --- | --- | --- |
| Number of years | 20 | 9 |
| Mean | 1.19 | 2.11 |
| p-value | 0.000 | |
| Standard deviation | 0.56 | 0.62 |
| Difference in mean | -0.92 | |
| Degree of freedom | 28 | |
| Effect size | -1.30 | |
| Alpha | 0.05 | |
| Beta | 0.03 | |
| Power | 0.97 | |

**
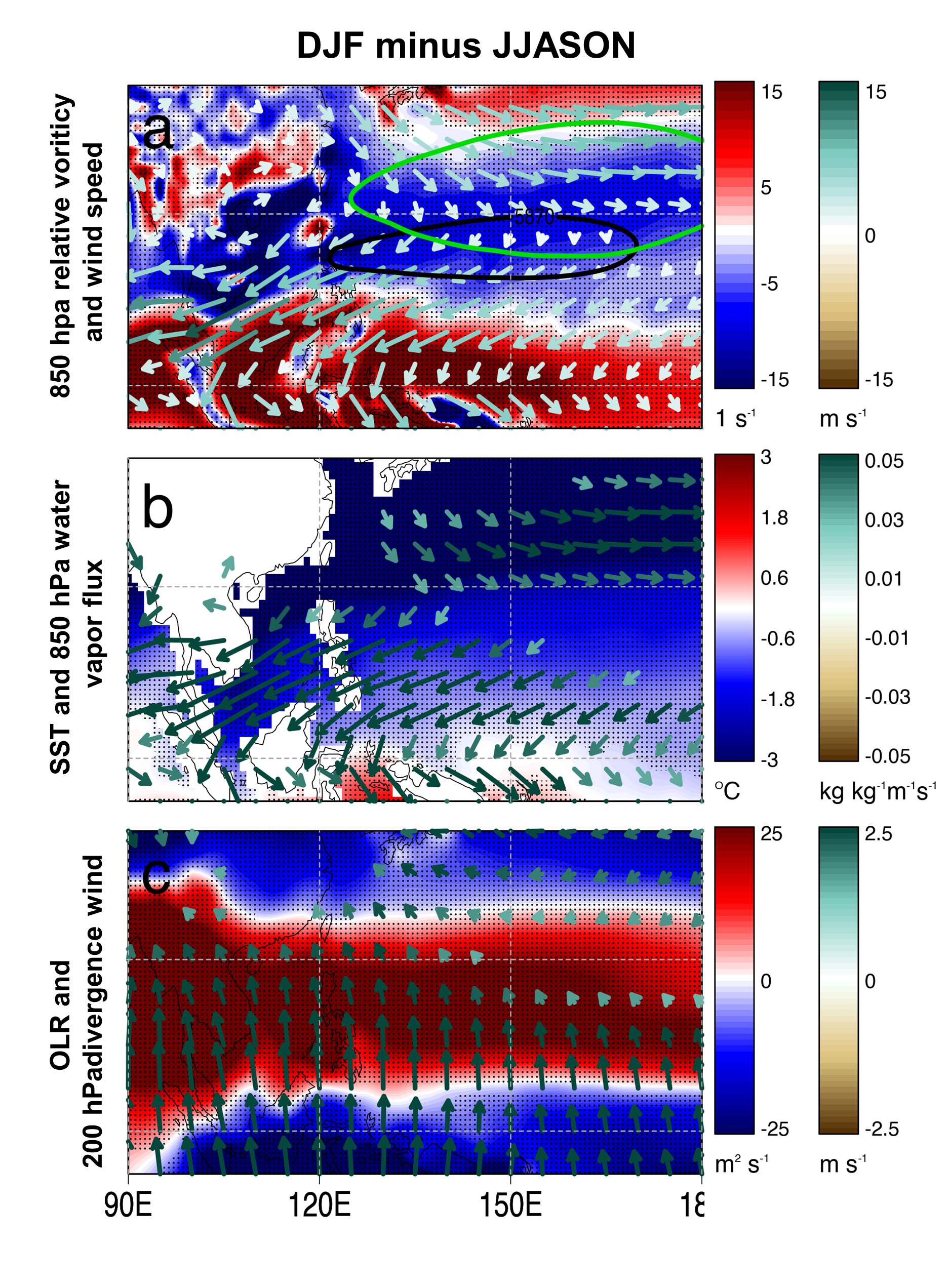
Supplementary Fig. S1.** **Composite analysis of indicated variables.** Composite difference between the less active season (December to February; DJF) and more active season (June to November) showing 850 hPa relative vorticity (shaded) and wind speed (vector) (**a**), sea surface temperature (shaded) and 850 hPa water vapor flux (**b**) and outgoing longwave radiation (shaded) and divergence wind (vector) (**c**). The magnitude of difference in the shaded and vector variables is shown in their respective scale bars. The black dots indicate significant difference at p<0.05 level, two-tailed. In **a**, the 850 hPa relative vorticity is multiplied with 1,000,000 for scaling while the black (green) contour represents the location of the Western North Pacific Subtropical High during the more (less) active season. In **a-c**, the maps are plotted using GrADS v2.2.1 (http://opengrads.org/).


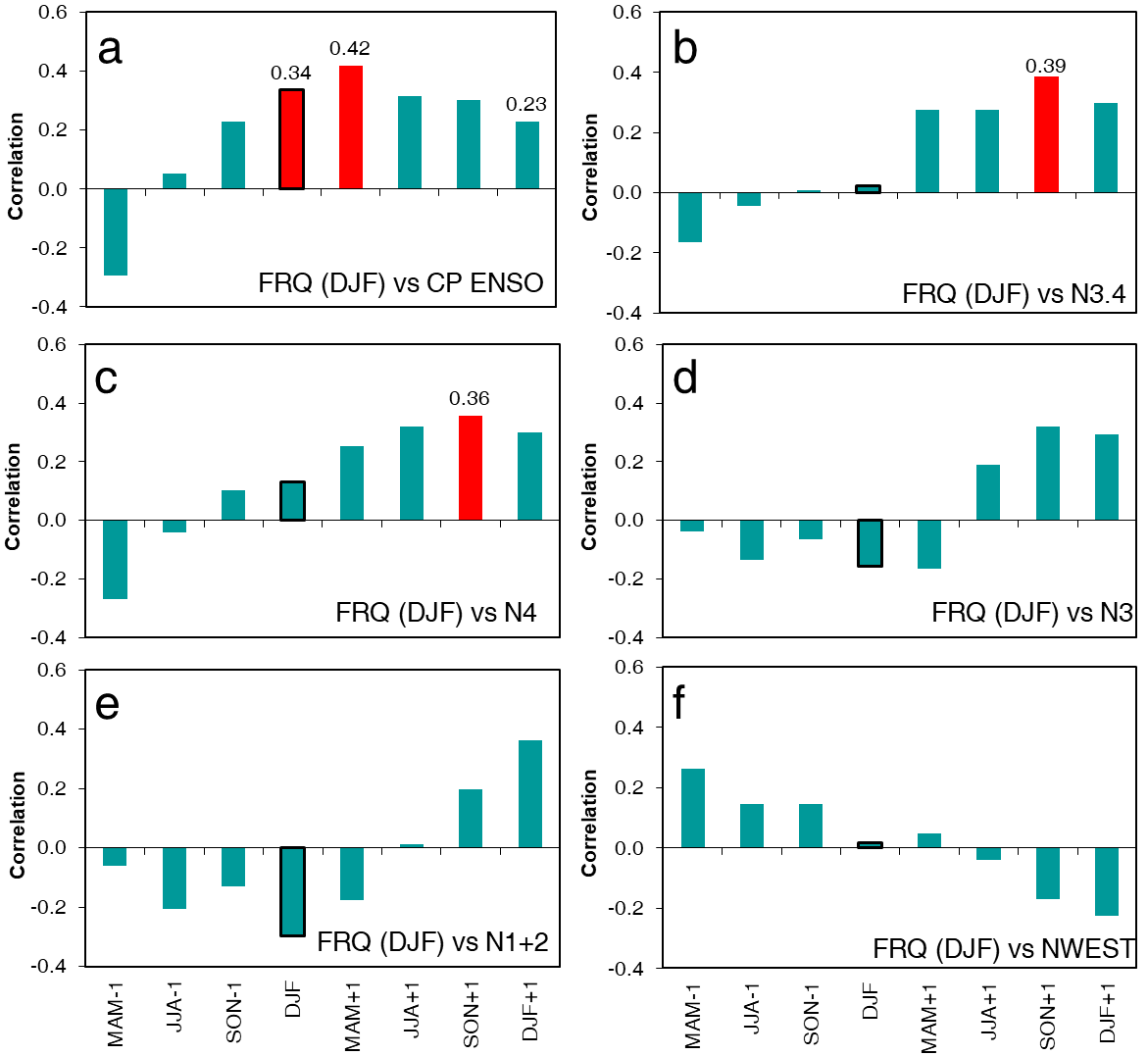


**Supplementary Fig. S2.** **Lead-lagged correlation of Christmas typhoons with different ENSO flavors.** The red bars indicate significant lead-lagged correlation (p<0.05, two-tailed) labeled with their corresponding coefficients.

**
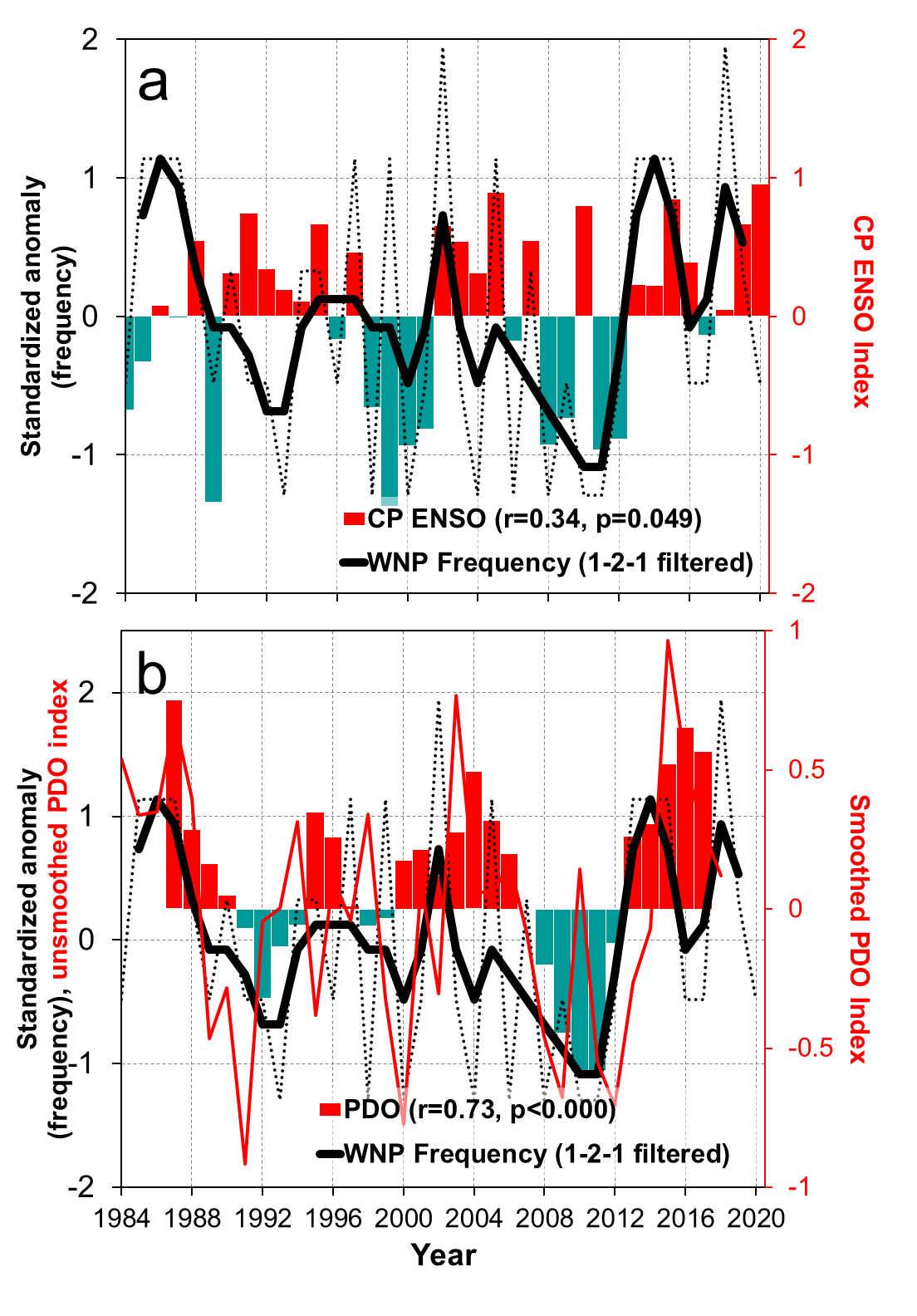
Supplementary Fig. S3.** **Timeseries of the Central Pacific ENSO (CP ENSO) and Pacific Decadal Oscillation (PDO).** **a,** CP ENSO and **b**, PDO. In **b**, the filled bar (red line) represents (unsmoothed) smoothed PDO using 7-year running centered. In **a-b**, the black dashed (solid) line indicates (1-2-1 filtered) Christmas typhoon frequency in the Western North Pacific.

**
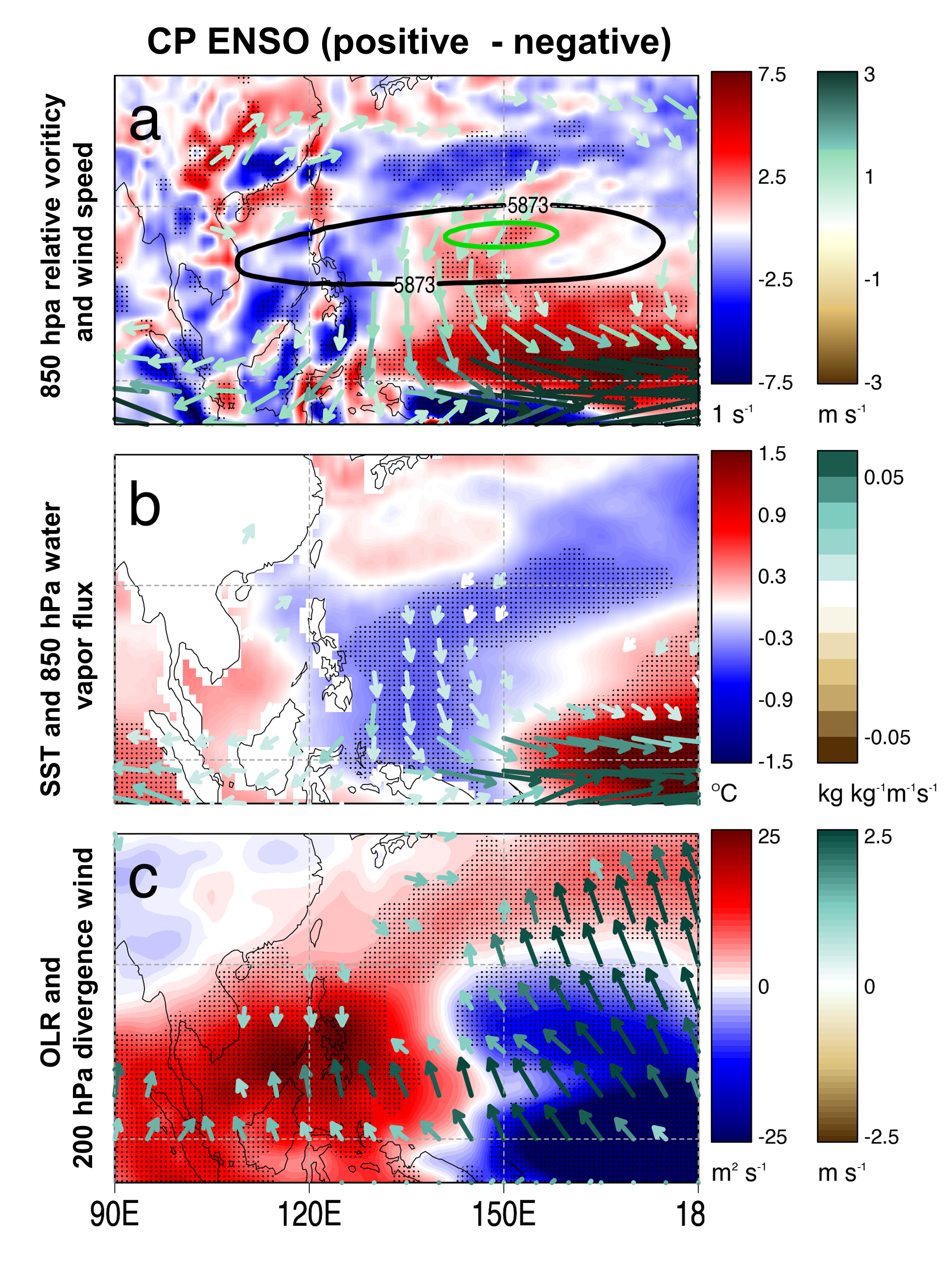
Supplementary Fig. S4.** **Composite difference of indicated variables between positive and negative CP ENSO years.** **a**, 850 hPa relative vorticity (shaded) and wind speed (vector). **b**, sea surface temperature (shaded) and 850 hPa water vapor flux (vector). **c**, outgoing longwave radiation (shaded) and 200 hPa divergence wind (vector). The black dots indicate significance at p<0.05 level. The magnitude of difference in the shaded and vector variables is shown in their respective scale bars. The black dots indicate significant difference at p<0.05 level. In **a**, the 850 hPa relative vorticity is multiplied with 1,000,000 for scaling while the black (green) contour represents the location of the Western North Pacific Subtropical High during the positive (negative) CP ENSO phase. In **a-c**, the maps are plotted using GrADS v2.2.1 (http://opengrads.org/).

**
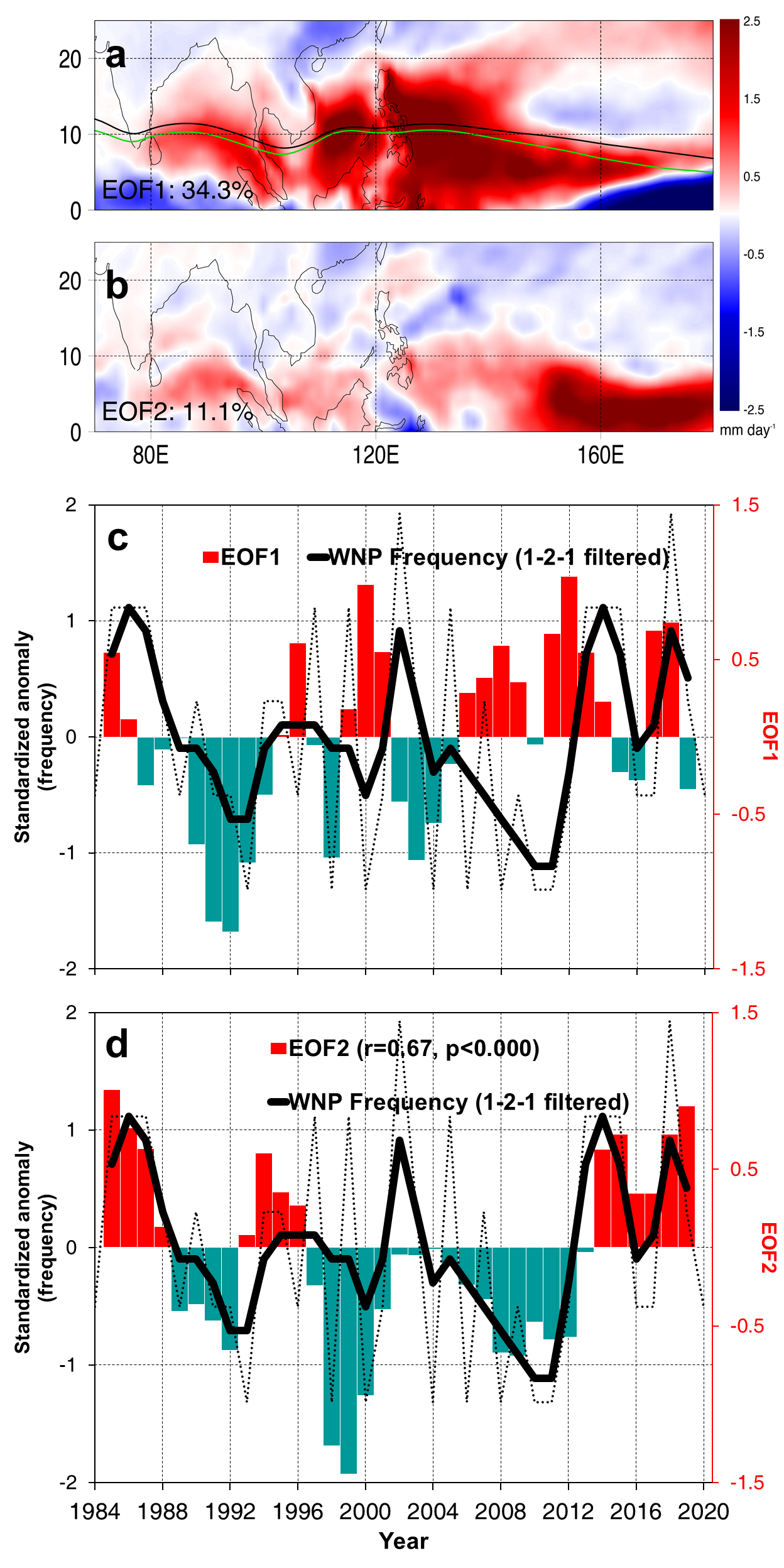
Supplementary Fig. S5.** **Influence of the Intertropical Convergence Zone (ITCZ) to Christmas typhoons.** **a-b**, The first and second leading Empirical Orthogonal Function (EOF) modes map of DJF precipitation, respectively. **c-d**, The time series of the principal components of the two leading EOF modes and Christmas typhoon frequency in the Western North Pacific, respectively. The black dashed (solid) line indicates (1-2-1 filtered) timeseries. In **a**, the black (green) contour line denotes the position of the DJF ITCZ during Period 2 (Period 1). In **a-b**, the maps are plotted using GrADS v2.2.1 (<http://opengrads.org/>)

**
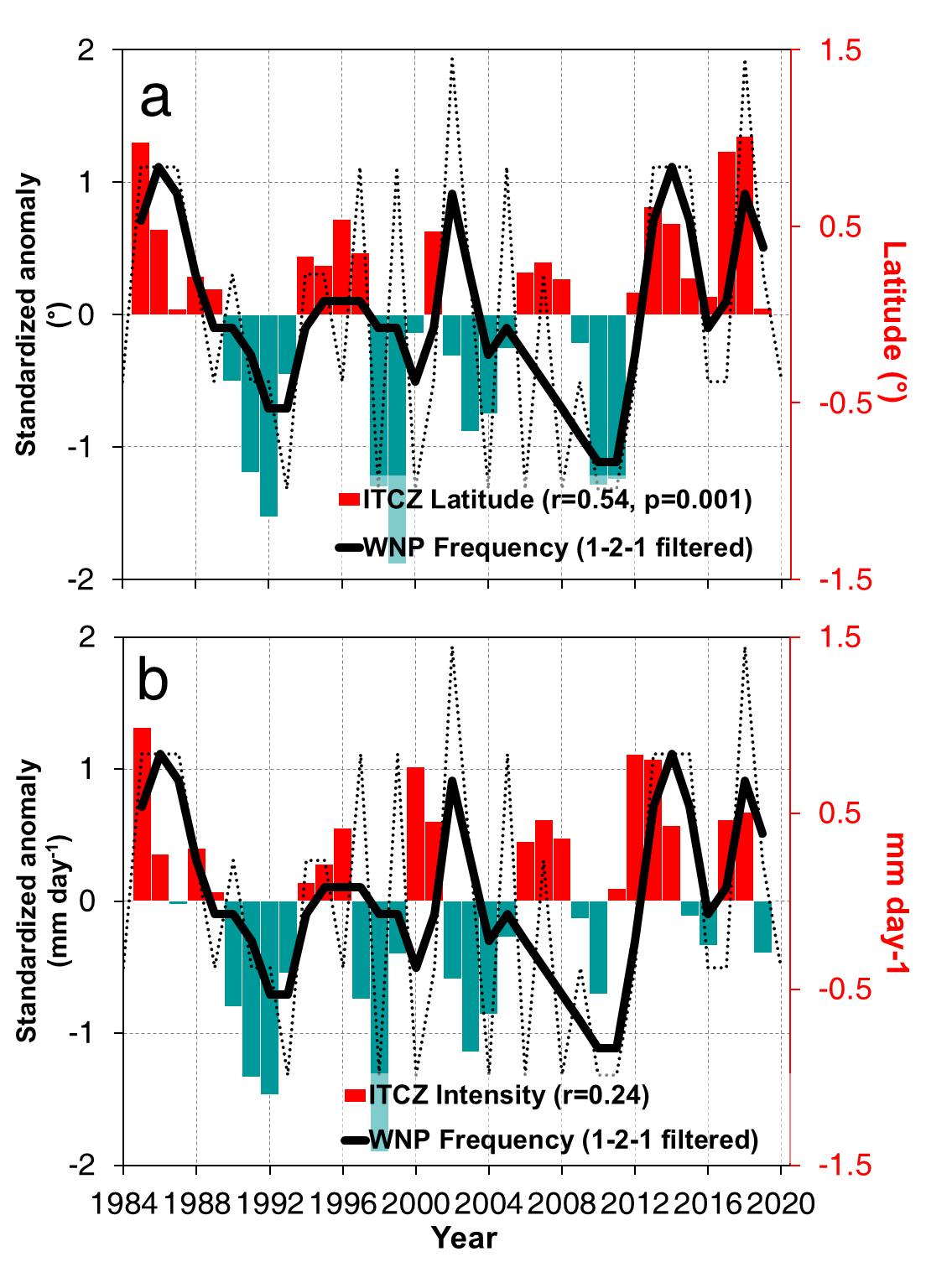
Supplementary Fig. S6. Timeseries of ITCZ metrics. a**, Standardized anomalies of the northern latitude of ITCZ latitude (bar) using zonal-averaged precipitation at 80-170°E during DJF in the Western North Pacific. **b**, Standardized intensity anomalies of the ITCZ using areal-averaged precipitation from 0-22°E, 80-170°E (bar). In **a-b,** the black dashed (solid) line indicates (1-2-1 filtered) timeseries.

**
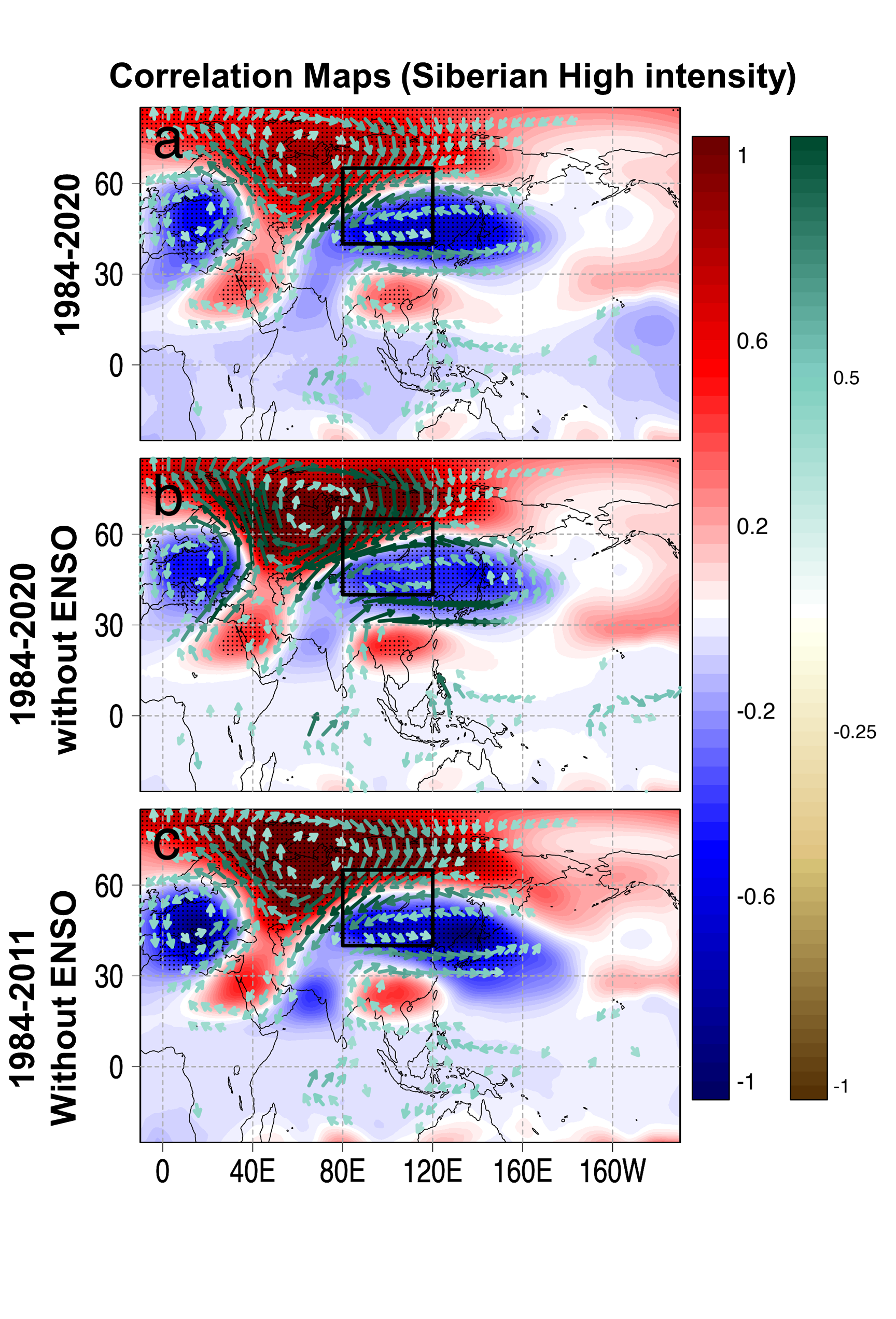
Supplementary Fig. S7.** **Influence of the Siberian High intensity (SHI) to tropical cyclones.** **a**, Correlation map of the DJF SHI index against DJF 400 hPa geopotential height and vector wind from 1984-2020. **b**, same with **a** but showing partial correlation map when the influence of DJF CP ENSO index is removed. **c**, same with **b** but from 1984-2011. The black dots indicate significance at p<0.05 level. The black box shows the location index of the Siberian High. In **a-c**, the maps are plotted using GrADS v2.2.1 (http://opengrads.org/).

**
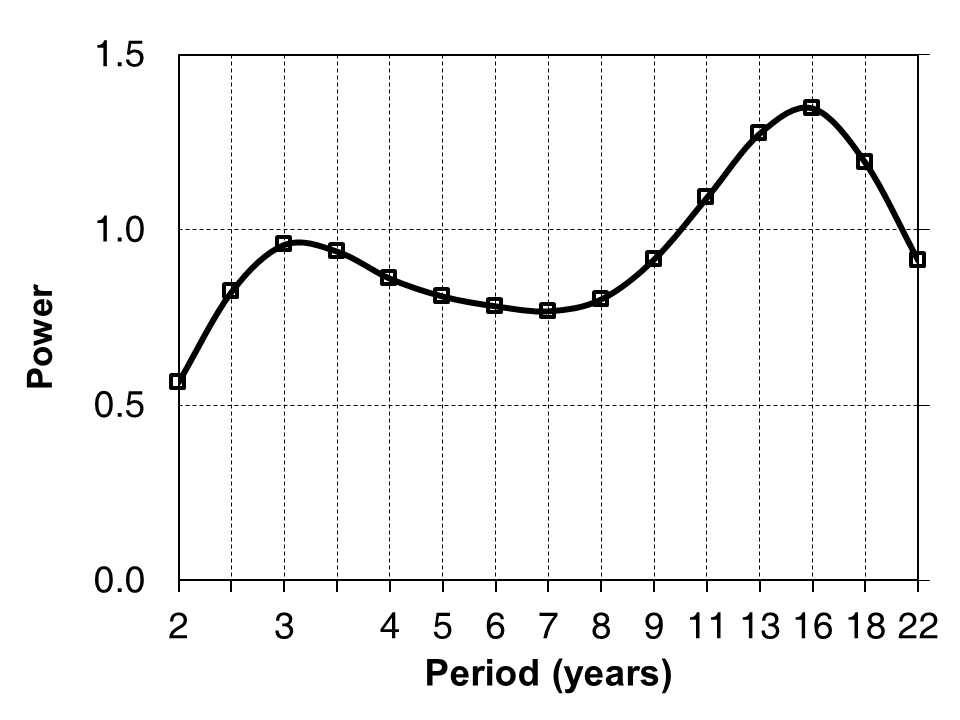
Supplementary Fig. S8.** **Paul wavelet transform of the timeseries of Christmas typhoons.**
